# The Non-Lantibiotic Bacteriocin Garvicin Q Targets Man-PTS in a Broad Spectrum of Sensitive Bacterial Genera

**DOI:** 10.1038/s41598-017-09102-7

**Published:** 2017-08-21

**Authors:** Aleksandra Tymoszewska, Dzung B. Diep, Paulina Wirtek, Tamara Aleksandrzak-Piekarczyk

**Affiliations:** 1 Institute of Biochemistry and Biophysics, Polish Academy of Sciences (IBB PAS), Pawińskiego 5a, 02-106 Warsaw, Poland; 2 Faculty of Chemistry, Biotechnology and Food Science, Norwegian University of Life Sciences, Ås, Norway

## Abstract

Mannose phosphotransferase system (Man-PTS) is the main mannose permease in bacteria but it is also a known receptor for subclass IIa bacteriocins (pediocin-like group) as well as subclass IId lactococcin A (LcnA) and lactococcin B (LcnB) (LcnA-like group). Subclass IIa bacteriocins exhibit a strong activity against *Listeria* spp. but they are not against *Lactococcus* spp. In contrast, the LcnA-like bacteriocins act only against *Lactococcus lactis* strains. Garvicin Q (GarQ) is a subclass IId bacteriocin with minor similarity to LcnA-like bacteriocins and a relatively broad antimicrobial spectrum including, among others, *Listeria* and *Lactococcus* spp. To identify the GarQ receptor, we obtained GarQ-resistant mutants of *Lactococcus garvieae* IBB3403 and *L*. *lactis* IL1403 and sequenced their genomes that revealed mutations in genes encoding the membrane-bound Man-PTS IIC or IID subunits encoded by *ptnCD* in *L*. *lactis* and *manCD* in *L*. *garvieae*. This is the first time that a bacteriocin outside the pediocin- and LcnA-like groups is shown to target Man-PTS. The interaction between GarQ and Man-PTS may occur through a new binding pattern involving specific amino acids highly conserved among the GarQ-sensitive bacterial species located in the N-terminal part and extracellular loops of subunit IID and in transmembrane region of IIC.

## Introduction

The emergence of antibiotic-resistant pathogens and an increasing consumer demand for more natural food preservatives have recently created an urgent need for new effective antimicrobials. One of the most promising groups of such compounds are bacteriocins, ribosomally synthesized antimicrobial peptides or proteins with a bactericidal or bacteriostatic activity. They are produced by both Gram-positive and Gram-negative bacteria, but those produced by Gram-positive bacteria, particularly lactic acid bacteria (LAB), show the greatest potential for application since bacteriocin-producing LAB strains can be used as starter and bioprotective cultures in the food industry^1^. The current classification of bacteriocins from Gram-positive bacteria divides them into two major classes: class I bacteriocins known as lantibiotics, which undergo post-translational modifications such as the generation of the thioether amino acids lanthionine and 3-methyllanthionine, and class II bacteriocins, so-called nonlantibiotics, which contain non-modified peptides or peptides with only minor modifications such as disulfide bridge or end-to-end circularization^2, 3^. The latter class has gained substantial interest due to their ubiquitous production by bacteria, great diversity in terms of amino acid sequence, physicochemical properties and target specificity, as well as width of inhibitory spectrum. The nonlantibiotics are further divided into (i) subclass IIa - pediocin-like bacteriocins, which are divided further into four (I-IV) groups and contain a conserved N-terminal amino acid sequence YGNGVXC, (ii) subclass IIb of two-peptide bacteriocins, (iii) subclass IIc of cyclic bacteriocins, and (iv) subclass IId of non-pediocin-like one-peptide bacteriocins^4, 5^.

The bacteriocins differ from each other by their inhibitory spectra, each targeting a defined set of bacteria, which suggests that they recognize specific receptors on the target cells^6^. However, the exact targets and molecular mechanisms underpinning this process are still almost unknown for most bacteriocins^2^. To date, only five bacteriocin receptors have been described for class II bacteriocins: (i) mannose phosphotransferase system (Man-PTS) recognized by all subclass IIa and a few subclass IId bacteriocins^7^; (ii) maltose ATP-Binding Cassette (ABC) transporter for garvicin ML, a circular subclass IIc bacteriocin^8^, (iii) the Zn-dependent metallopeptidase YvjB for the subclass IId LsbB^9^, (iv) undecaprenyl pyrophosphate phosphatase (UppP) for the subclass IIb lactococcin G^10^, and (v) a putative amino acid transporter for the subclass IIb plantaricin JK^11^.

Man-PTS belongs to the family of phosphoenolpyruvate dependent sugar transporters and can simultaneously import and phosphorylate selected monosaccharides, such as mannose, glucose, glucosamine or fructose. Man-PTS consists of the cytoplasm-located components IIA and IIB, which often occur as a single polypeptide chain, and transmembrane subunits IIC and IID, through which the sugars are transported^12^. Early studies showed that Man-PTS, apart from its principal sugar-transporter function, could also serve as a receptor for subclass IIa bacteriocins. For example, *Listeria monocytogenes* mutants resistant to pediocin-like bacteriocins (pediocin PA-1, leucocin A) displayed reduced expression of the Man-PTS-encoding *mptACD* operon^13, 14^. Moreover, inactivation of the *mptACD* operon in *L*. *monocytogenes* and *Enterococcus faecalis* caused resistance to mesentericin Y105^15, 16^. Conversely, the heterologous expression of the *mptACD* operon in insensitive *Lactococcus lactis* strains conferred sensitivity to leucocin A, pediocin PA-1 and enterocin A^17^. The studies above provide strong genetic evidence between the receptor genes and the associated sensitivity to the bacteriocins when the receptor genes are intact and expressed. However, a direct evidence of a contact between the receptor and the bacteriocin was still missing in these studies. This conclusive piece of evidence came from a study by Diep *et al*., 2007 who showed by immuno-precipitation, a direct interaction between the Man-PTS complex and a bacteriocin upon its binding to target cells, and further delimited the receptor functions to the membrane-located components IIC and IID of the complex^7^. Surprisingly, also two subclass IId bacteriocins, LcnA and LcnB, unrelated to the subclass IIa bacteriocins, have been reported to use Man-PTS subunits IIC and IID as a receptor^7^.

The Man-PTSs can be divided into several phylogenetic groups and so far only members of phylogenetic group I have been shown to serve as receptors for class II bacteriocins. The group I Man-PTSs contain three distinct regions termed α, β and γ localized in the IIC (α and β) and IID (γ) subunits^6^. Region α is indispensable for specific binding of subclass IIa bacteriocins and has been found to be present in the sensitive *L*. *monocytogenes* strains and absent in the resistant *L*. *lactis* strains. A distinct spectrum of activity has been observed for Man-PTS-targeting subclass IId bacteriocins LcnA and LcnB active only against *L*. *lactis* species^18^.

GarQ is a subclass IId bacteriocin produced by *Lactococcus garvieae* BCC 43578 isolated from fermented-pork sausage^19^. It uses a so called double-glycine leader for secretion and the mature peptide is 50 amino acids long. GarQ has a fairly broad antimicrobial spectrum being active against several species from the genera *Bacillus*, *Enterococcus*, *Lactobacillus*, *Lactococcus* and *Pediococcus*. Despite lacking the YGNGVXC motif characteristic for pediocin-like bacteriocins, GarQ is also active against *L*. *monocytogenes* ^19^.

In this study we report the identification of the receptor for garvicin Q, which turned out to be the IIC and IID subunits of Man-PTS. We show that despite recognizing the same receptor on the same host cells (*L*. *lactis*), GarQ and LcnA-group bacteriocins differ in their mode of interaction with Man-PTS. This difference may involve specific interactions between the bacteriocin and individual amino acids localized in the Man-PTS subunits IIC and IID. We experimentally identify the amino acids of Man-PTS from *L*. *lactis* and *L*. *garvieae* potentially responsible for GarQ binding and show that they are not involved in the binding of LcnA-group bacteriocins. The same seven amino acids are required for GarQ binding in both *L*. *garvieae* and *L*. *lactis* Man-PTS. Most of these amino acids are conserved in Man-PTSs from other GarQ-sensitive species, suggesting a similar mode of interaction with GarQ. These data indicate that across bacterial species Man-PTS may constitute an important target specifically recognized by multiple non-homologous bacteriocins.

## Materials and Methods

### Bacterial strains and culture conditions

The bacterial strains used in this study are listed in Table S1 in the Supplementary File. Indicator strains used to determine the activity spectrum of a bacteriocin, strains used to obtain spontaneous GarQ-resistant mutants, and *L*. *lactis* IL1403 with a deletion or complementation of the Man-PTS-encoding operon were grown in brain heart infusion (BHI) medium (Oxoid, Hampshire, United Kingdom) at 30 °C. *Lactococcus* spp. used to obtain spontaneous GarQ-resistant mutants with missense mutations were grown in chemically defined medium (CDM)^20^ supplemented with 1% mannose (man-CDM) at 30 °C. *Escherichia coli* EC1000 was grown in Luria-Bertani (LB) medium (Becton, Dickinson and Company, New Jersey, United States) at 37 °C. To grow *E*. *coli* EC1000 with pNZ8037 plasmid, chloramphenicol was added to 20 µg/ml and to grow *L*. *lactis* IL1403 with pNZ9530 and pNZ8037 plasmids, erythromycin and chloramphenicol to 5 µg/ml each were added to the growth medium. Transcription of the *ptnCD* genes cloned in pNZ8037 under the nisin-responsive promoter (Supplementary Table S1) was induced by the addition of nisin to between 0.1 and 10 ng/ml. Sensitivity to bacteriocins was determined by an inhibitory spectrum assay as described below. Soft agar (soft BHI-agar, soft man-CDM-agar) and agar plates (BHI-agar, man-CDM-agar) were prepared by adding agar (Merck, Darmstadt, Germany) to the final concentration of 0.75% and 1.5%, respectively, to appropriate liquid media.

### Bacteriocin preparation

Bacteriocin in the form of lyophyllisate with a purity of over 95% was obtained by a commercial chemical synthesis (PepMic, Suzhou, China). Before use, peptide was dissolved to the required concentration in 0.1% trifluoroacetic acid (TFA) (Sigma, Darmstadt, Germany).

### Inhibitory spectrum assay

To assess the activity spectrum of a bacteriocin, indicator bacteria were inoculated and grown overnight (o/n) in BHI liquid medium, then 100 µl of the o/n culture was used to inoculate 5 ml of soft BHI-agar pre-heated to 55 °C. The molten agar was immediately poured onto a BHI-agar plate and distributed evenly. Following agar setting, 5 µl of suitable bacteriocin dilutions (0.03; 0.06; 0.125; 0.25; 0.5; 1 mg/ml) was spotted on selected locations on the plate. The plates were allowed to dry and were incubated o/n at 30 °C.

### Selection of GarQ-resistant mutants

GarQ-resistant mutants of *L*. *garvieae* IBB3403 and *L*. *lactis* IL1403 were collected from small colonies that occasionally appeared in the inhibition zones during inhibitory spectrum assay or were selected in a dedicated experiment. For this purpose to 5 ml of soft BHI-agar or soft man-CDM-agar containing 100 µl of an indicator o/n-culture, 100 µl of suitable bacteriocin dilutions (as above) was added and the plates were incubated for single colonies to appear. Bacterial species identity was confirmed by 16 S rDNA sequencing using primers 27 F and 1492 R (Supplementary Table S1). To determine the level of resistance of the isolated GarQ-resistant mutants they were grown on microtiter plates with serial two-fold bacteriocin dilutions. The assays were performed in triplicate. The minimum inhibitory concentration value (MIC50) was determined as the lowest concentration of bacteriocin that resulted in reduction of bacterial growth by more than 50%. Stability of the resistance phenotype was confirmed by passaging the bacteria on plates without bacteriocin and then repeating the microtiter plate assay.

### Genomic DNA isolation, DNA sequencing and data analysis

DNA was isolated using Genomic Mini kit (A&A Biotechnology, Gdynia, Poland). Samples for genome sequencing were prepared using Nextera XT DNA Sample Preparation kit, Nextera XT Indexing kit (96-indexes) and PhiX control V3 kit and sequenced on a Miseq Sequencer (Illumina, San Diego, Ca, United States). For each strain about 1.5–2.5 million 250-nt paired-end reads were obtained. The data were analyzed with CLC Genomics Workbench 6.5.2 (Qiagen, Hilden, Germany) and DNAStar SeqMan Gen (DNAStar, Madison, Wi, United States). Samples for *manABCD* operon (from *L*. *garvieae*) sequencing were prepared by PCR on genomic DNA with primers *manC*for/rev and *manD*for/rev (Supplementary Table S1). Samples for *ptnABCD* operon (from *L*. *lactis*) sequencing were prepared by PCR on genomic DNA with primers *ptnC*for/rev and *ptnD*for/rev (Supplementary Table S1). Amplified DNA was purified using Wizard® SV Gel and PCR Clean-Up System (Promega, Fitchburg, Wi, United States). To translate a nucleotide sequence to protein sequence, the translate tool on the ExPasy online server was used^21^. To compare protein sequences, the Clustal Omega software at the EMBL-EBI online server^22^ or MultAlin software^23^ were used. For prediction of transmembrane protein regions, the HMMTOP server was used^24^. Predicted topology of the membrane Man-PTSs subunits was visualized using Protter^25^. To predict a secondary GarQ structure, the fully automated protein structure homology-modelling SWISS-MODEL server was used^26^.

### Phenotypic profiling and growth tests

Fermentation patterns of 49 sugars under anaerobic conditions were determined using the API 50 CH test (BioMerieux, Marcy l’Etolie, France) as specified by the manufacturer and recorded after 24, 48 and 72 h of incubation at 30 °C. Growth tests were performed in M17 medium (Oxoid, Hampshire, United Kingdom) supplemented with 1% cellobiose (cel-M17), 1% glucose (glu-M17) or 1% mannose (man-M17), using Microbiology Reader Analyser (Bioscreen C; Oy Growth Curves Ab Ltd, Helsinki, Finland). Optical density at 600 nm was monitored during 72 h of incubation at 30 °C in 1-h intervals. Assays were performed in triplicate. Exponential growth rates were determined as previously described^27^. The ability to use mannose as a single carbon source was determined by growing bacteria in CDM medium supplemented with 1% mannose (man-CDM).

### Site-directed mutagenesis

Recombinant pNZ8037 plasmid with the *ptnCD* genes has been obtained previously^7^, whereas the one with the *manCD* genes was created in this study by ligation of the pNZ8037 vector with a PCR product containing the *manCD* genes, amplified with primers compl*manCD*for/rev and digested with NcoI and XhoI. These recombinant pNZ8037 plasmids carrying either *ptnCD* or *manCD* genes were transformed into *E*. *coli* EC1000 resulting in the *E*. *coli* B529a or *E*. *coli* B530a strains, respectively. In the next step, recombinant pNZ8037 plasmids with the *ptnCD* or *manCD* genes were isolated using PureYield^TM^ plasmid mini-prep system (Promega, Fitchburg, Wi, United States) and used as template in site-directed mutagenesis, which was performed according to the Cornell iGEM 2012 Protocol (http://2012.igem.org/wiki/images/a/a5/Site_Directed_Mutagenesis.pdf). In order to introduce point mutations Pro102His or Ala104Val into *ptnC*, mutagenic primers *ptnC*Pro102Hisfor/rev or *ptnC*Ala104Valfor/rev, respectively, were used. To introduce respective point mutations into *ptnD*, the mutagenic primers *ptnD*Pro108Serfor/rev, *ptnD*Thr120Ilefor/rev or *ptnD*Gly244Valfor/rev, were used. To introduce the Pro126His point mutation into *manD*, mutagenic primers *manD*Pro126Hisfor/rev were used. All primer sequences are shown in Table S1 in the Supplementary File.

## Results

### GarQ has a relatively wide activity spectrum

Although the GarQ activity spectrum has been examined previously by others^19^, in this study we reexamined it using a larger panel of microorganisms. GarQ showed a wide inhibitory spectrum being active against all tested bacterial species from the genera *Carnobacterium*, *Enterococcus*, *Lactococcus*, *Leuconostoc* and *Listeria* (Table 1). In the case of the *Lactobacillus* and *Pediococcus* genera, the susceptibility to GarQ was species-specific. *Lactobacillus kunkeei* and *Pediococcus acidilactici* showed resistance to GarQ, while the other *Lactobacillus* and *Pediococcus* spp. tested were sensitive to GarQ. Among the GarQ sensitive species tested, *E*. *faecalis*, *Enterococcus faecium*, *Lactobacillus rhamnosus*, *L*. *garvieae* and *L*. *lactis* strains were the most sensitive. GarQ showed no activity against *Candida albicans*, *Pseudomonas aeruginosa*, *Salmonella typhimurium* or all the tested species of the genera *Bacillus*, *Campylobacter*, *Staphylococcus* and *Streptococcus*.

**Table 1:** Inhibitory spectrum of GarQ and bacterial ability to utilize mannose.

Indicator strain	Sensitivity to GarQ	Growth on mannose
*Bacillus cereus* IBB3390	−	−
*Bacillus subtilis* 168	−	−
*Campylobacter jejuni* 12	−	−
*Campylobacter jejuni* 480	−	−
*Campylobacter jejuni* 81176	−	−
*Campylobacter coli* 23	−	−
*Candida albicans* CAI-4	−	−
*Carnobacterium maltaromaticum* IBB3447	+	+
*Enterococcus durans* IBB3441	+	+
*Enterococcus faecalis* IBB3439	++	+
*Enterococcus faecalis* IBB3444	++	+
*Enterococcus faecalis* LMGT 2003	+	+
*Enterococcus faecium* LMGT 2783	++	+
*Enterococcus faecium* LMGT 2787	+	+
*Lactobacillus casei* IBB3418	+	+
*Lactobacillus casei* IBB3427	+	+
*Lactobacillus casei* LOCK 0919	+	+
*Lactobacillus casei* IBB3423	+	+
*Lactobacillus casei/paracasei* IBB3425	+	+
*Lactobacillus casei/paracasei* IBB3426	+	+
*Lactobacillus casei/paracasei* IBB3428	+	+
*Lactobacillus paracasei* IBB3424	+	+
*Lactobacillus johnsonii* IBB3155	+	+
*Lactobacillus kunkeei* AH1	−	−
*Lactobacillus kunkeei* AH38	−	−
*Lactobacillus kunkeei* AH119	−	−
*Lactobacillus paraplantarum* IBB3438	+	+
*Lactobacillus plantarum* NC8	+	+
*Lactobacillus plantarum* WCSF1	+	+
*Lactobacillus plantarum* IBB3036	+	+
*Lactobacillus plantarum* IBB3433	+	+
*Lactobacillus plantarum* IBB3436	+	+
*Lactobacillus plantarum* IBB3434	+	+
*Lactobacillus rhamnosus* IBB3429	+	+
*Lactobacillus rhamnosus* LOCK 0900	++	+
*Lactobacillus rhamnosus* LOCK 0908	++	+
*Lactobacillus rhamnosus* GG	+	+
*Lactobacillus salivarius* IBB3154	+	+
*Lactococcus garvieae* IBB3403	++	+
*Lactococcus garvieae* IBB66	++	+
*Lactococcus lactis* IBB3404	++	+
*Lactococcus lactis* IBB3411	++	+
*Lactococcus lactis* QU5 LMGT 3419	+	+
*Lactococcus lactis* subsp. *cremoris* IBB3409	++	+
*Lactococcus lactis* subsp. *lactis* IL1403	++	+
*Lactococcus lactis* subsp. *lactis* IBB2955	++	+
*Lactococcus lactis* subsp. *lactis* IBB3407	++	+
*Lactococcus raffinolactis* IBB91	+	+
*Leuconostoc lactis* IBB3446	+	+
*Leuconostoc mesenteroides* IBB3442	+	+
*Leuconostoc mesenteroides* IBB3443	+	+
*Listeria monocytogenes* EGD-e	+	+
*Pediococcus acidilactici* LMGT 2002	−	−
*Pediococcus parvulus* IBB3448	+	+
*Pseudomonas aeruginosa* ATCC 9027	−	−
*Salmonella typhimurium* TT622	−	−
*Staphylococcus aureus* ATCC 6538	−	−
*Staphylococcus caprae* DSM-20608	−	−
*Staphylococcus delphini* DSM-20771	−	−
*Staphylococcus epidermidis* DSM-20044	−	−
*Staphylococcus hyicus* DSM-20459	−	−
*Staphylococcus intermedius* DSM-20373	−	−
*Staphylococcus lugdunensis* DSM-4804	−	−
*Staphylococcus pseudintermedius* DSM-21284	−	−
*Staphylococcus saprophyticus* DSM-18669	−	−
*Staphylococcus schleiferi* DSM-6628	−	−
*Streptococcus agalactiae* IBB123	−	−
*Streptococcus agalactiae* IBB130	−	−
*Streptococcus mitis* IBB3449	−	−
*Streptococcus sobrinus* IBB3450	−	−
*Streptococcus parauberis* IBB272	−	−

“−” indicates that no inhibition zone was observed, “+” indicates that an inhibition zone in diameter less or equal to 10 mm was observed, while “++” indicates that an inhibition zone in diameter larger than 10 mm was observed.

### Selection of *L*. *garvieae* IBB3403 GarQ-resistant mutants

To identify genes involved in the sensitivity to GarQ in target cells that could encode a GarQ receptor, we isolated resistant mutants of the highly sensitive strain *L*. *garvieae* IBB3403 by exposing the cells to increasing concentrations of GarQ on BHI-agar plates. Twenty-one independent spontaneous GarQ-resistant mutants were obtained at GarQ concentration of 1 mg/ml (mutants PW200 - PW220; Table 2). All these mutants were equally resistant to GarQ with the MIC value over 1024-fold higher than that of the parental *L*. *garvieae* IBB3403 (MIC = 0.098 µg/ml). After multiple passages without selective pressure of GarQ, no loss of resistance was observed indicating that the resistance was due to stable genetic mutations rather than an adaptation event.

**Table 2:** Spontaneous *L*. *garvieae* IBB3403 and *L*. *lactis* IL1403 mutants resistant to GarQ.

Mutant strain	Mutation	Amino acid sequence change	Position of affected amino acid(s)	Level of resistance to GarQ	Growth on mannose
*L*. *garvieae* IBB3403					
PW200	C304 → T in *manD*	Gln102 → STOP	outside	1024x	−
PW201	T314 → A in *manC*	Leu105 → STOP	transmembrane	1024x	−
PW202	C299 → A in *manC*	Pro100 → His	transmembrane	1024x	+
PW203	C368 → T in *manD*	Thr123 → Ile	outside	1024x	+
PW204 LGN8 LGN10	C331 → T in *manD*	Pro111 → Ser	outside	1024x	+
PW205	G4 → T in *manD*	Ser2 → STOP	outside	1024x	−
PW206	C410 → A in *manC*	Ser137 → STOP	inside	1024x	−
PW207 PW209	G499 → T in *manC*	Glu167 → STOP	transmembrane	1024x	−
PW208	insertion 511 A in *manD*	frameshift at Thr171	inside	1024x	−
PW210	G86 → A in *manD*	Trp29 → STOP	outside	1024x	−
PW211 PW213	C104 → A in *manC*	Ser35 → STOP	transmembrane	1024x	−
PW212	deletion 391 G in *manC*	frameshift at Ala131	inside	1024x	−
PW214 PW219	C102 → A in *manC*	Cys34 → STOP	transmembrane	1024x	−
PW215	C70 → T in *manC*	Gln24 → STOP	outside	1024x	−
PW216	C145 → T in *manD*	Gln9 → STOP	outside	1024x	−
PW217	C727 → T in *manD*	Gln243 → STOP	outside	1024x	−
PW218	G49 → T in *manC*	Gly17 → STOP	transmembrane	1024x	−
PW220	C153 → A in *manD*	Tyr51 → STOP	outside	1024x	−
LGN1 LGN5 LGN7	G902 → T in *manD*	Gly301 → Val	outside	1024x	+
LGN2 LGN3	C398 → T in *manD*	Ala133 → Val	transmembrane	16x	+
LGN4	G767 → T in *manD*	Trp256 → Leu	outside	16x	+
LGN6	G800 → T in *manD*	Gly267 → Val	outside	16x	+
LGN9	C305 → T in *manC*	Ala102 → Val	transmembrane	16x	+
*L*. *lactis* IL1403					
AT1	insertion 545AT in *ptnC*	frameshift at Ile182	transmembrane	1024x	−
AT2 AT6	794 *ptnC* - 51 *ptnD* → insertion of a chromosomal fragment	frameshift at D265 in IIC, loss of 20 aa in IID	outside	1024x	−
AT3	deletion 323 C in *ptnD*	frameshift at Pro108	outside	1024x	−
AT4	TTGTTGTTG35 → AGGATA in *ptnC*	IleValValAla11 → LysAspThr	transmembrane	1024x	−
AT5	insertion 532 A in *ptnD*	frameshift at Leu178	transmembrane	1024x	−
AT7	insertion 559CT in *ptnC*	frameshift at Ala187	transmembrane	1024x	−
AT8	deletion 433TTC in *ptnD*	deletion of Phe145	transmembrane	1024x	−
AT9	insertion 550 T in *ptnD*	frameshift at Lys184	transmembrane	1024x	−
AT10	insertion 525 G in *ptnD*	frameshift at Gly175	transmembrane	1024x	−
LLN1-LLN10	C368 → A in *ptnD*	Pro123 → His	outside	1024x	+

### GarQ-resistant mutants contain mutations in genes encoding Man-PTS

**Figure 1:**
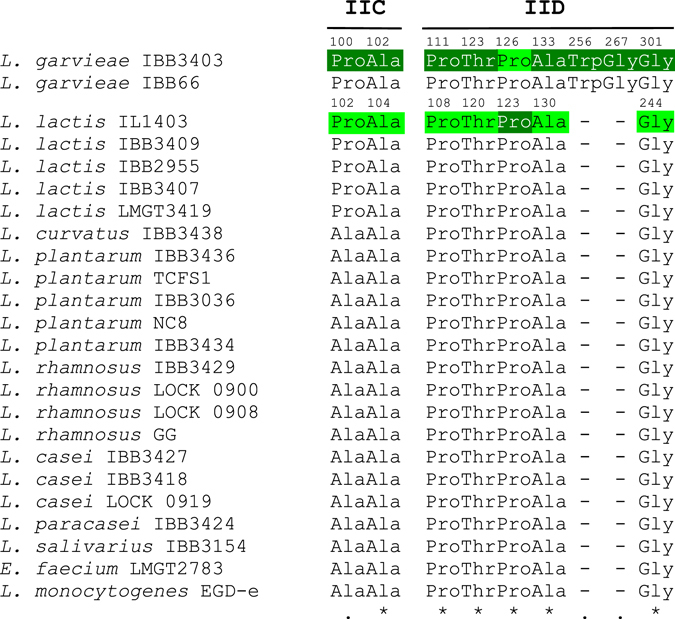
Alignment of Man-PTS IIC and IID *L*. *garvieae* IBB3403 and *L*. *lactis* IL1403 substituted amino acids (missense mutations) in different species. Amino acid substitutions (missense mutations) introduced by spontaneous mutation or by site-directed mutagenesis are highlighted dark green or light green, respectively, and their counterparts in other species are not highlighted. Numbers indicate positions of substituted amino acids in *L*. *garvieae* IBB3403 and *L*. *lactis* IL1403. Asterisks indicate fully conserved residues, single dots - weakly conserved residues.

**Figure 2:**
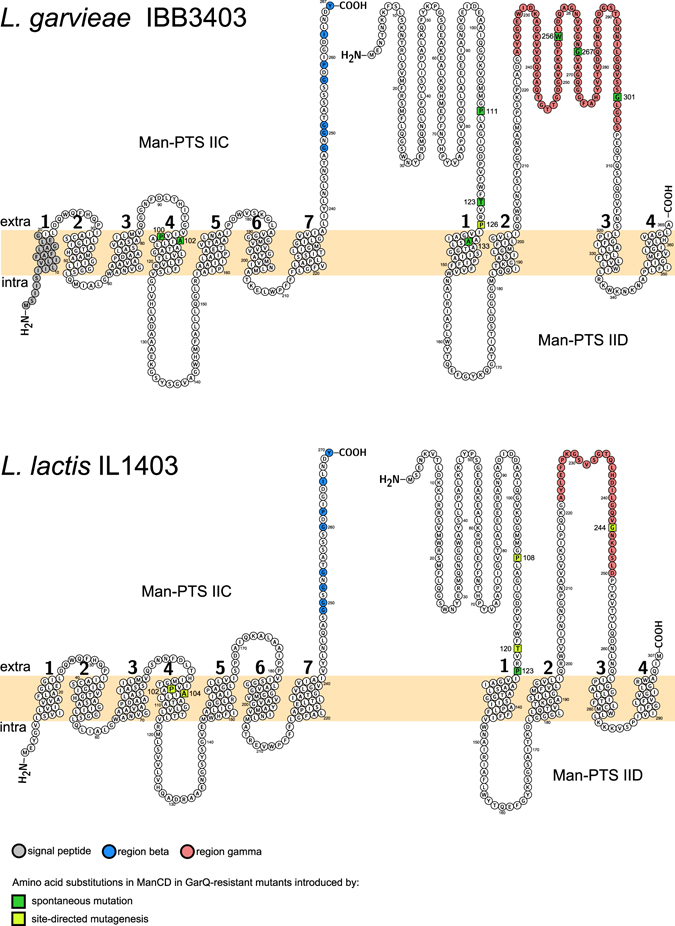
Predicted topology of the membrane Man-PTS subunits IIC and IID of *L*. *garvieae* IBB3403 and *L*. *lactis* IL1403.

In order to identify the mutations in the genomes of the GarQ-resistant *L*. *garvieae* IBB3403 strains, DNA was isolated from nine randomly selected spontaneous mutants (PW200 - PW205, PW207, PW209 and PW210), sequenced and compared with the genome of parental GarQ-sensitive *L*. *garvieae* IBB3403. Single mutations were detected exclusively in the *manABCD* operon encoding Man-PTS (Table 2). Encouraged by this result, we performed direct sequencing of the *manABCD* operon in the remaining twelve GarQ-resistant mutants using specific primers. Altogether, among the twenty-one sequenced mutants, twelve harbored mutations in the *manC* gene encoding the IIC subunit, while the remaining nine mutants contained mutations in the *manD* gene coding for the IID subunit (Table 2). No mutation was found in the *manAB* gene encoding the cytoplasmic Man-PTS IIAB component, suggesting that this component is likely not required for the sensitivity to GarQ. The *manCD* mutations were of different types. Most caused premature truncation of the IIC or IID polypeptides, due to either nonsense or frameshift mutations in the *manC* or *manD* genes, and only three were missense mutations. Remarkably, in all three missense mutants the substituted amino acids were in the predicted outer regions of the transmembrane subunits IIC or IID. The PW202 mutant carried a C299 → A mutation in *manC* resulting in Pro100 → His substitution, while the PW203 and PW204 mutants carried single C368 → T and C331 → T mutations in *manD* resulting in Thr123 → Ile and Pro111 → Ser substitutions, respectively (Figs 1 and 2; Supplementary Fig. S1).

To assess whether the involvement of Man-PTS in GarQ sensitivity is species specific, we performed similar mutagenesis in the model LAB organism *L*. *lactis* IL1403. By the same approach, ten spontaneous *L*. *lactis* IL1403 mutants resistant to GarQ were obtained at 1 mg/ml GarQ (AT1 - AT10; Table 2) and all had the MIC values more than 1024-fold higher than the parental strain (0.024 µg/ml). Sequencing of their *ptnABCD* operons encoding Man-PTS revealed mutations in genes encoding the IIC or IID subunits and none in the gene coding for the IIAB component. The majority of the mutations were frameshift ones and none of the mutants harboured a missense mutation (Table 2).

### Both IIC and IID subunits are indispensable for GarQ activity

**Figure 3:**
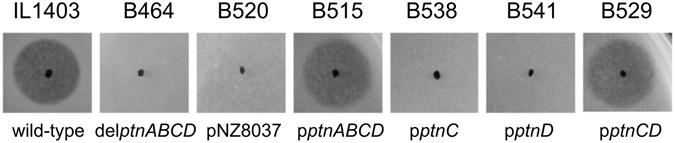
Sensitivity of the *L*. *lactis* clones to garvicin Q. IL1403 (wild-type strain), B464 (*ptnABCD* deletion strain), B520 (*ptnABCD* deletion strain expressing empty pNZ8037 plasmid), B515, B538, 541, B529 (*ptnABCD* deletion strains expressing *ptnABCD*, *ptnC*, *ptnD* and *ptnCD*, respectively).

The sequencing data strongly suggested that the observed GarQ resistance was due to the mutations in the *ptnC* or *ptnD* genes; however, a role of some unidentified mutations outside the *ptnABCD* operon could not be ruled out. To find out whether a compromised Man-PTS is sufficient to confer GarQ resistance we checked the GarQ sensitivity of an *L*. *lactis* strain missing the entire *ptnABCD* operon, *L*. *lactis* B464^7^. This *ptnABCD*-deletion strain had a MIC value ca. 1024-fold higher than the wild-type *L*. *lactis* IL1403, indicating full GarQ resistance. To identify the Man-PTS subunit(s) required for GarQ sensitivity, we performed a complementation test with different combinations of the three genes comprising the *ptnABCD* operon^7^ (Supplementary Table S1). As expected, the complete *ptnABCD* operon complemented the *L*. *lactis* B464 defect and restored GarQ sensitivity. No single gene (*ptnAB*, *ptnC*, or *ptnD*) alone was able to confer GarQ sensitivity, but two genes combined, *ptnC* and *ptnD*, were sufficient to cause GarQ sensitivity similar to that of a wild-type strain (Fig. 3). Altogether, these results clearly show that both IIC and IID subunits are required and sufficient for sensitivity to GarQ, whereas IIAB is not.

### Garvicin Q structure, its similarity to other Man-PTS-binding bacteriocins and inhibitory spectrum

**Figure 4:**
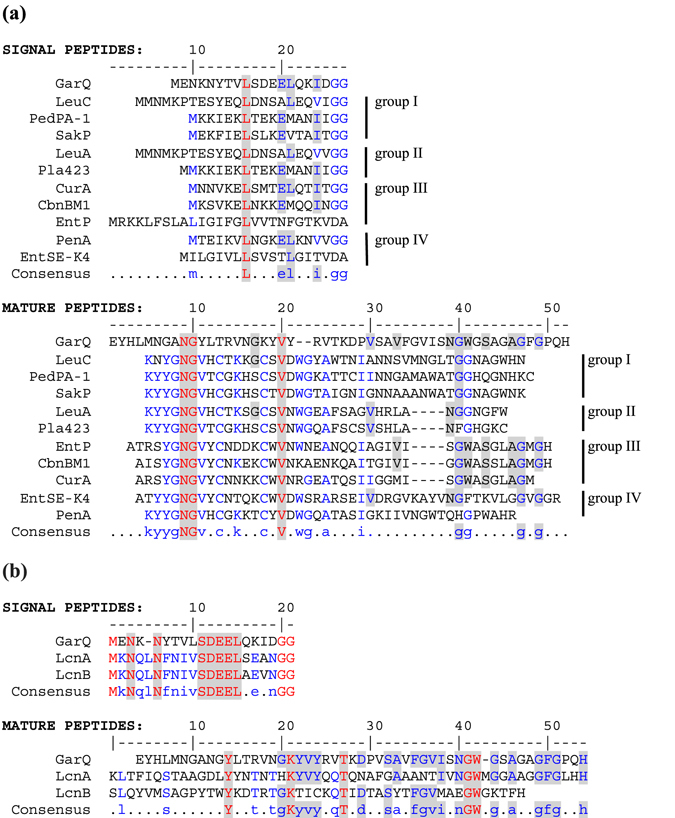
Multiple sequence alignment of the amino acid sequences of signal peptides and mature peptides of garvicin Q and class IIa (pediocin-like) (**a**) and LcnA-like class IId (**b**) bacteriocins targeting Man-PTS. Full consensus and low consensus amino acids are indicated with red and blue letters, respectively. In a bacteriocin and consensus sequence the amino acids conserved among class IIa or LcnA-like class IId bacteriocins are not shaded, whereas the ones conserved between GarQ and class IIa or LcnA-like class IId bacteriocins are highlighted grey. The UniProt accession numbers are P38579 for carnobacteriocin BM1 (CbnBM1), P0A311 for curvacin A (CurA), O30434 for enterocin P (EntP), C9B989 for enterocin SE-K4 (EntSE-K4), H6U5Y1 for garvicin Q, P0A313 for lactococcin A (LcnA), P35518 for lactococcin B (LcnB), P34034 for leucocin A (LeuA), H2EST2 for leucocin C (LeuC), P29430 for pediocin PA-1 (PedPA-1), M1GJX1 for penocin A (PenA), Q93FV7 for plantaricin 423 (Pla423) and P35618 for sakacin P (SakP).

Man-PTS has been shown to be the receptor for IIa pediocin-like and two IId LcnA-like bacteriocins^7^, hence, we compared the GarQ amino acid sequence with these bacteriocins, to assess their relatedness. For this comparison we chose 2–3 members of each of four pediocins-like groups. Leader peptides of GarQ and pediocins showed almost no similarity (Fig. 4a). Mature peptides of GarQ and three group III pediocins - carnobacteriocin BM1, curvacin A and enterocin P showed some similarity at their C-terminal end (such as conservation of the GWxSxxAG motif) but mature peptides of GarQ and pediocins of the other tested subclasses showed almost no similarity (Fig. 4a). In contrast, the leader and mature peptides of GarQ and LcnA-like bacteriocins are more similar to each other, especially between the mature peptides of GarQ and LcnA (Fig. 4b).

A comparison of the activity spectra of the Man-PTS-targeting bacteriocins showed that GarQ, with its broad spectrum, is clearly distinct from the others (Table 1) since pediocin-like and LcnA-like bacteriocins are only active against *Listeria* spp. and *L*. *lactis*, respectively^18^. Since no information was available on the activity of the pediocin-like bacteriocins and LcnA against *L*. *garvieae* strains, we assayed the sensitivity of *L*. *garvieae* IBB3403 to sakacin A, pediocin PA1 and LcnA. These three bacteriocins showed no activity against *L*. *garvieae* IBB3403. Such specific inhibitory spectra suggest distinct modes of interaction of GarQ, LcnA and pediocin-like bacteriocins with their Man-PTS targets.

Next, based on partial templates available from Protein Data Bank (PDB), the secondary structure of GarQ was predicted to have an unstructured N-termini, whereas the C-terminal part was α-helical. Moreover, protein sequence homology searches revealed that the 30 amino acids sequence of GarQ C-terminal part shares around 50% identity and 69% similarity with sodium:proline solute transporter.

### Sugar fermentation pattern of GarQ-resistant mutants depends on the nature of mutation

Given that GarQ targets a sugar transport system, we undertook to examine how the acquisition of resistance to GarQ affected the bacterial sugar metabolism. We profiled sugar utilization by selected *L*. *garviaeae* GarQ-resistant mutants containing missense or nonsense mutations in *manCD*. For the 49 sugars tested, the only specific defect was the inability of the nonsense mutant strains PW205 and PW210 to ferment mannose; their glucose metabolism remained intact.

**Figure 5:**
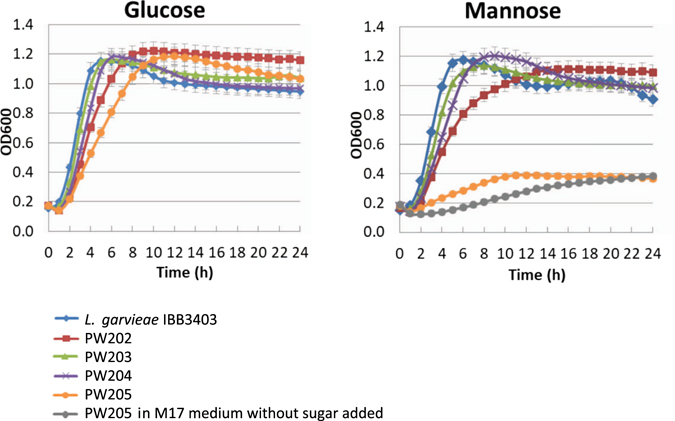
Growth of wild-type *L*. *garvieae* IBB3403 and *L*. *garvieae* IBB3403 spontaneous mutants resistant to GarQ in M17 medium supplemented with glucose or mannose. PW202 carries Pro100 → His substitution in subunit IIC, PW203 Thr123 → Ile substitution in IID, PW204 Pro111 → Ser substitution in IID and PW205 nonsense mutation in IID. As a control growth of PW205 in M17 medium without sugar added is shown. Data are presented as mean ± SD (n = 3).

To investigate the sugar metabolism of the GarQ-resistant mutants in more detail, their growth kinetics was determined in M17 medium supplemented with the Man-PTS-transported glucose or mannose, or with cellobiose not using Man-PTS for transport. In cel-M17, all mutants grew with the same kinetics as the wild-type strain (data not shown), whereas in glu- and man-M17 strain- or sugar-dependent growth differences were detected (Fig. 5). In glu-M17, all GarQ-resistant mutants exhibited slightly lower growth rates (between 0.480 h^−1^ for PW203 and 0.287 h^−1^ for PW205) than the wild-type *L*. *garviaeae* IBB3403 strain (0.562 h^−1^). In man-M17, mutants with missense mutations in *manD* (*L*. *garviaeae* PW203 and PW204) exhibited no significant differences in growth rate, while the PW202 mutant with a missense mutation in *manC* displayed a slightly lower growth rate (0.221 h^−1^) than the wild-type (0.462 h^−1^). The most significant decrease of the growth rate (0.042 h^−1^) and the final optical density was observed for the PW205 mutant harboring a nonsense mutation in *manD* (Fig. 5). Its growth was equal to the residual growth (in M17 medium without any sugar), indicating a total inability to metabolize mannose. Further tests to assess the ability to grow on man-CDM-agar plates of the remaining GarQ-resistant mutants confirmed the mannose-negative phenotype of the nonsense and frameshift mutants and mannose-positive of the missense ones (Table 2).

### Resistance of bacterial species to GarQ is connected with their inability to utilize mannose

To determine whether species resistant to GarQ (Table 1) harbor genes encoding Man-PTS, we inspected their genomes available in the GenBank database. No genes encoding phylogenetic group I Man-PTSs were found in the genomes of *Bacillus cereus*, *Bacillus subtilis*, *Campylobacter coli*, *Campylobacter jejuni*, *C*. *albicans*, *L*. *kunkeei*, *P*. *aeruginosa*, *S*. *typhimurium* and *Staphylococcus* spp., which explains their resistance to GarQ (Table 1). In contrast, the genomes of *P*. *acidilactici*, and *Streptococcus* spp. carried entire operons encoding phylogenetic group I Man-PTSs, despite these species being resistant to GarQ. To estimate whether these Man-PTSs are functional, we tested these bacteria for growth on man-CDM. None of the GarQ-resistant species could grow on the man-CDM medium (Table 1) indicating that even the presence of an operon encoding a putative Man-PTS does not enable the bacteria to utilize mannose as a sole carbon source. This suggests that the putative Man-PTS-encoding operons were not functional, albeit one could envisage a situation where the bacterium would take up mannose using Man-PTS but would be then unable to metabolize it.

We also determined that species sensitive to GarQ harbored genes encoding phylogenetic group I Man-PTSs and manifested mannose-positive phenotype (Table 1). Taken together these results indicate a direct dependency between species’ ability to use mannose as a carbon source (functional or non-functional Man-PTS) and their sensitivity to GarQ.

### Specific amino acids of Man-PTS are required for sensitivity to GarQ

Approach described in a previous study^7^ showed by immuno-precipitation a direct interaction between the Man-PTS complex and a bacteriocin upon its binding to target cells, however, was not conclusive about receptor specific amino acids that may be involved in a contact with a bacteriocin. Method developed in this study and a subsequent analysis of the isolated GarQ-resistant mutants pinpointed regions and individual amino acids of Man-PTS that are required for the *L*. *lactis* and *L*. *garvieae* sensitivity to GarQ and thus are presumably involved in the interaction with this bacteriocin. We based on the observation that, in contrast to the GarQ-resistant nonsense or frameshift mutants, the GarQ-resistant missense mutants maintained the mannose-positive phenotype. Thus, using a medium containing 1 mg/ml GarQ and mannose as a sole carbon source (man-CDM-GarQ) we selected *L*. *lactis* IL1403 and *L*. *garvieae* IBB3403 GarQ-resistant mutants that had a mannose-positive phenotype and harbored missense mutations in their respective Man-PTS-encoding operons. The obtained *L*. *lactis* IL1403 GarQ-resistant mutants were over 1024-fold more resistant than the parental strain, while their sensitivity to LcnA was unaffected, which further underlined the distinct modes of interaction of these two bacteriocins with Man-PTS. Among the *L*. *garvieae* isolates, five were over 1024-fold more resistant to GarQ than the parental strain and five – only ca. 16-fold. We then sequenced the relevant *ptnABCD* and *manABCD* operons from the ten obtained *L*. *lactis* IL1403 and ten *L*. *garvieae* IBB3403 GarQ-resistant mutants. Unexpectedly, all ten independent *L*. *lactis* mutants (viz. LLN1-LLN10) had the same C368 → A transition of a single nucleotide in *ptnD* resulting in the Pro123 → His substitution in the outer N-terminal region of the IID subunit of the Man-PTS (Table 2; Figs 1 and 2; Supplementary Fig. S1).

The ten independent *L*. *garvieae* GarQ-resistant mutants carried six different single mutations: one in *manC* resulting in the Ala102 → Val substitution (LGN9) and five in *manD* resulting in the Pro111 → Ser (LGN8, LGN10), Ala133 → Val (LGN2, LGN3), Trp256 → Leu (LGN4), Gly267 → Val (LGN6) or Gly301 → Val (LGN1, LGN5, LGN7) substitutions (Table 2; Figs 1 and 2; Supplementary Fig. S1). The substituted amino acids were localized either in the outer regions of Man-PTS subunit IID (*L*. *garvieae* LGN1, LGN4-LGN8, LGN10) or in the fourth (*L*. *garvieae* LGN9) or first (*L*. *garvieae* LGN2, LGN3) transmembrane regions of subunits IIC or IID, respectively (Figs 1 and 2; Supplementary Fig. S1). The Pro111 → Ser and Gly301 → Val mutants were over 1024-fold less sensitive to GarQ and the others 16-fold (Table 2).

At this point we also tested two *L*. *lactis* IL1403 LcnA-resistant mutants: *L*. *lactis* LMGT3726 with a missense mutation in *ptnD* resulting in the Pro115 → His substitution in IID, and *L*. *lactis* LMGT3727 with a missense mutation in *ptnC* resulting in the Gly184 → Asp substitution in IIC^10^. Both these mutants were still sensitive to GarQ, once again indicating that distinct amino acids of Man-PTS are responsible for the interaction with the GarQ and LcnA bacteriocins.

### The amino acids altered by missense mutations in GarQ-resistant strains are conserved among GarQ-sensitive bacterial species

To examine whether the amino acids found to be required for *Lactococcus* spp. sensitivity to/binding GarQ are conserved among the other GarQ-sensitive species, we sequenced the operons encoding phylogenetic group I Man-PTSs of the eleven strains used in the activity spectrum assay. Additionally, we inspected sequences of eight phylogenetic group I Man-PTSs from genomes deposited in GenBank (their accession numbers are listed in Table S1 in the supplementary file). Six of the nine amino acids that were substituted in the GarQ-resistant mutants isolated in the present study (Ala102 in *L*. *garvieae* IIC, Pro111, Thr123, Ala133, Gly301 in *L*. *garvieae* IID and Pro123 in *L*. *lactis* IID) were strictly conserved in all the GarQ-sensitive species/strains inspected (Fig. 1; Supplementary Fig. S1). The other substituted amino acids (Pro100 in *L*. *garvieae* IIC and Trp256 and Gly267 in *L*. *garvieae* IID) were conserved only among the *Lactococcus* genus and *L*. *garvieae* strains, respectively. In other species, an alanine was found instead of Pro100 present in *Lactococcus* spp. The last two amino acids, Trp256 and Gly267, required for *L*. *garvieae* IBB3403 sensitivity to GarQ, had no equivalents in the other species as they are present in an additional extracellular loop of IID specific to *L*. *garvieae* (Fig. 2; Supplementary Fig. S1).

Since different amino acids were altered by missense mutations in GarQ-resistant mutants of *L*. *garvieae* IBB3403 and *L*. *lactis* IL1403 (Table 2), we tested whether mutating the same amino acids in these two species would make them insensitive to GarQ. Thus, by site-directed mutagenesis we introduced missense mutations that appeared by random mutagenesis in *ptnABCD* into *manABCD* and vice-versa, and expressed the mutated operons in *L*. *lactis* B464 with a full deletion of the *ptnABCD* operon. All the strains obtained by this procedure (Supplementary Table S1) turned out to be less sensitive to GarQ than the reference *L*. *lactis* B529 or *L*. *lactis* B530a strains indicating the all the substituted amino acids are required for GarQ toxicity, and presumably also for GarQ-Man-PTS interaction in the two *Lactococcus* species.

## Discussion

Pathogenic *L*. *garvieae* strains may cause bovine mastitis^28^ and lactococcosis, a hyperacute systemic disease that can occur in cultured or wild fish species^29^. Thus, bacteriocins produced by, and active against, *L*. *garvieae* strains show an applicative potential in the prevention or treatment of these infections. Besides, like other bacteriocins produced by Gram-positive bacteria, they can be used to combat certain infections caused by Gram-positive pathogens other than *L*. *garvieae*. To date, six bacteriocins produced by *L*. *garvieae* have been reported: garviecin L1-5^30^, garvicin ML^31^, garvicin Q^19^, garvicin A^32^, garviecin LG34^33^ and garvicin KS^34^. However, receptors for most of these bacteriocins on target cells remain unknown. Here, we chose GarQ, which has a broad activity spectrum with genus or species-directed specificity. Importantly, GarQ is active against *Listeria* species infecting humans and animals, *L*. *monocytogenes* the etiological agent of listeriosis, and *Listeria ivanovii* causing gastroenteritis and bacteremia, which suggests its possible therapeutic use.

To identify the GarQ receptor, we used whole genome sequencing (WGS) of spontaneous GarQ-resistant mutants. Such an approach has been demonstrated to be appropriate for genotypic analysis of resistant mutants^9–11^. The consistent pattern of mutations identified in numerous independently derived spontaneous GarQ-resistant strains pointed to Man-PTS being required for susceptibility to GarQ. This conclusion was verified experimentally. Deletion of the operon encoding Man-PTS and subsequent phenotypical studies confirmed that a functional Man-PTS is essential for bacterial sensitivity to GarQ. Furthermore, complementation with genes encoding only the membrane components of Man-PTS (IIC and IID) confirmed that both these subunits are needed for GarQ sensitivity, whereas the cytoplasmic IIAB component is dispensable.

Man-PTS is a system indispensable for mannose uptake by a bacterial cell^12^. Phenotypic tests of the GarQ-resistant mutants obtained during this study revealed a strong correlation between the type of the mutation and the ability to metabolize mannose. Mutants with nonsense or frameshift mutations in genes encoding Man-PTS were no longer able to metabolize mannose, while those harboring missense mutations were still able to grow on mannose (Table 2). The deficiency of mannose metabolism in a select group of GarQ-resistant mutants suggests that their nonsense or frameshift mutations had a deleterious effect on Man-PTS structure, which deprived it of its crucial mannose-uptake function. On the other hand, the retained ability to utilize mannose by the missense GarQ-resistant mutants suggests that the amino acid substitutions in these mutants had no negative effect on the transmembrane structure of Man-PTS preserving its functionality related to sugar transport. In line with this observation is the mannose fermentation deficiency of all GarQ-resistant indicator species (Table 1), suggesting that in these bacteria Man-PTS-encoding operons are absent or non-functional. Indeed, we could not identify such operons in some of GarQ-resistant species’ genomes, while in other species a potential Man-PTS-encoding operon was clearly present. We believe that in the latter cases operons were non-functional as these cells were not only insensitive to GarQ but also unable to exploit mannose as a carbon source. The latter due to the fact that Man-PTS is the only permease that facilitates import of mannose into bacterial cells. Our comparative analyses of the *man-PTS* ribosome binding site (RBS) spacer length (the distance between the initiation codon and the RBS) revealed extended sequences (10–11 nucleotides) in GarQ-resistant and mannose-negative species, whereas shorter ones (7–9 nucleotides) in GarQ-sensitive and mannose-positive species. Thus, it is plausible that the occurrence of longer RBS spacers above *man-PTS* may be a factor that negatively affects the formation of the 30 S initiation complex leading to the severe reduction or lack of the Man-PTS proteins synthesis resulting in GarQ-resistant and mannose-negative phenotype. The efficient use of glucose by those strains was likely due to the fact that for glucose, in contrast to mannose, also other sugar transporting systems can be used.

A comparison of amino acid sequences and activity spectra of GarQ with other bacteriocins that use Man-PTS as a receptor on target cells revealed that GarQ shares no or weak similarity with these bacteriocins. It is known that pediocins are active only against strains containing region α in the Man-PTS IIC subunit and therefore they act against *L*. *monocytogenes* but not against *L*. *lactis* ^18^. Conversely, LcnA-like bacteriocins are only active against *L*. *lactis* strains^18^. Interestingly, GarQ targets both *L*. *monocytogenes* and *L*. *lactis* strains, and is also active against *L*. *garvieae* strains, which do not have a fully conserved region α in their Man-PTS IIC subunits. As we showed in this study, pediocin- and LcnA-like bacteriocins show no activity against *L*. *garvieae* strains. This suggests that GarQ must interact with its receptor via a mechanism distinct from the mechanisms by which other bacteriocins recognize Man-PTS. Further support for this hypothesis comes from the fact that the *L*. *lactis* IL1403 mutants with a missense mutation in the *ptnD* gene altering Pro123 in subunit IID, were fully resistant to GarQ but were still sensitive to LcnA. Reciprocally, two *L*. *lactis* IL1403 LcnA-resistant strains with missense mutations substituting Gly188 in IIC or Pro115 in IID subunit of Man-PTS were still sensitive to GarQ. These data clearly indicate that distinct amino acids of the Man-PTS subunits IIC and IID are engaged in the interactions with GarQ, LcnA and pediocin-like bacteriocins.

Moreover, we found that in the missense GarQ-resistant mutants, the level of resistance to GarQ varied from 16- to over 1024-fold compared to the wild-type lactococcal strains (Table 2). This difference depended on the localization of the amino acid substituted and may be explained by diversified binding affinities between GarQ and the specific IICD amino acids. Thus, it is tempting to propose that the amino acids whose substitutions caused the high resistance to GarQ (Pro100 in *L*. *garvieae* IIC, Thr123, Pro111 and Gly301 in *L*. *garvieae* IID, and Pro123 in *L*. *lactis* IID) are the main determinants of the GarQ binding. Other amino acids, substitutions of which only led to partial resistance to GarQ (Ala102 in *L*. *garvieae* IIC and Ala133, Trp256 and Gly267 in *L*. *garvieae* IID), likely have only a supporting role in the GarQ - Man-PTS interaction (Fig. 2). All these Man-PTS amino acids potentially interacting with GarQ seem to be easily accessible for the bacteriocin, as most of them are localized in predicted outer regions of Man-PTS, and the rest in transmembrane regions adjacent to the outer membrane face (Fig. 2).

In this study we confirmed that among *Lactococcus* species the same set of Man-PTS amino acids is necessary for sensitivity to/binding with GarQ. Moreover, these amino acids, with a single exception, are highly conserved also among other GarQ-sensitive species (Supplementary Fig. S1). This conservation suggests that in all GarQ-sensitive species the same amino acids of Man-PTS are involved in the interaction with GarQ.

It is tempting to speculate that among the GarQ-sensitive species, the N-terminal part of the bacteriocin initially interacts with, depending on the species, five or seven amino acids of the Man-PTS IID extracellular loops specified in this study. This in turn may enable interaction of the C-terminal α-helix-containing part of the bacteriocin with two IIC amino acids (proline and alanine). As these two amino acids are predicted to be transmembrane-localised, we can further assume that they are part of the IIC inner pore helix. Such GarQ-receptor interaction might trigger structural changes in Man-PTS arousing the IIC permease to open as a pore, thereby leading to leakage of solutes across the membrane, disruption of membrane integrity and finally cell death. We can also presume that due to the similarity of the GarQ C-terminus to transporter domain, GarQ molecules bound to IIC may form a stable pore, which, by its structure, might act to facilitate the leakage of solutes.

Altogether, our results show that the presence of phylogenetic group I Man-PTS determines the sensitivity of genera *Carnobacterium*, *Enterococcus*, *Lactobacillus*, *Lactococcus*, *Leuconostoc*, *Listeria* and *Pediococcus* to garvicin Q. To the best of our knowledge, GarQ is the first bacteriocin outside the pediocin- or LcnA-like bacteriocins shown to interact with Man-PTS. It is also the first bacteriocin targeting Man-PTSs from both *L*. *monocytogenes* and *L*. *lactis*. GarQ interacts with Man-PTS through a new, previously unknown binding pattern involving specific amino acids located in the extracellular loops and N-terminal end of the IID subunit and in a transmembrane region of the subunit IIC. This study presents a deeper insight into the important role of Man-PTS as the receptor for multiple unrelated bacteriocins. Our further studies will focus on understanding how the distinct bacteriocins recognize the same receptor and how Man-PTS and bacteriocins co-evolved to allow such promiscuity^35–51^.

## Electronic supplementary material

Supplementary Table S1 & Figure S1

## References

[1] Jack RW, Tagg JR, Ray B (1995). Bacteriocins of gram-positive bacteria. Microbiol. Rev..

[2] Kjos M (2011). Target recognition, resistance, immunity and genome mining of class II bacteriocins from gram-positive bacteria. Microbiol. Read. Engl..

[3] Nes, I. F., Gabrielsen, C., Brede, D. A. & Diep, D. B. Novel developments in bacteriocins from lactic acid bacteria. in *Biotechnology of Lactic* Acid *Bacteria* (eds Mozzi, F., Raya, R. R. & Vignolo, G. M.) 80–99, doi:10.1002/9781118868386.ch5 (John Wiley & Sons, Ltd, 2015).

[4] Cotter PD, Hill C, Ross RP (2005). Bacteriocins: developing innate immunity for food. Nat. Rev. Microbiol..

[5] Cui Y (2012). Class IIa bacteriocins: diversity and new developments. Int. J. Mol. Sci..

[6] Kjos M, Nes IF, Diep DB (2009). Class II one-peptide bacteriocins target a phylogenetically defined subgroup of mannose phosphotransferase systems on sensitive cells. Microbiol. Read. Engl..

[7] Diep DB, Skaugen M, Salehian Z, Holo H, Nes IF (2007). Common mechanisms of target cell recognition and immunity for class II bacteriocins. Proc. Natl. Acad. Sci..

[8] Gabrielsen C, Brede DA, Hernández PE, Nes IF, Diep DB (2012). The maltose ABC transporter in *Lactococcus lactis* facilitates high-level sensitivity to the circular bacteriocin garvicin ML. Antimicrob. Agents Chemother..

[9] Uzelac G (2013). A Zn-dependent metallopeptidase is responsible for sensitivity to LsbB, a class II leaderless bacteriocin of *Lactococcus lactis* subsp. *lactis* BGMN1-5. J. Bacteriol..

[10] Kjos M (2014). Sensitivity to the two-peptide bacteriocin lactococcin G is dependent on UppP, an enzyme involved in cell-wall synthesis. Mol. Microbiol..

[11] Oppegård C, Kjos M, Veening J-W, Nissen-Meyer J, Kristensen T (2016). A putative amino acid transporter determines sensitivity to the two-peptide bacteriocin plantaricin JK. MicrobiologyOpen.

[12] Postma PW, Lengeler JW, Jacobson GR (1993). Phosphoenolpyruvate:carbohydrate phosphotransferase systems of bacteria. Microbiol. Rev..

[13] Ramnath M, Beukes M, Tamura K, Hastings JW (2000). Absence of a putative mannose-specific phosphotransferase system enzyme IIAB component in a leucocin A-resistant strain of *Listeria monocytogenes*, as shown by two-dimensional sodium dodecyl sulfate-polyacrylamide gel electrophoresis. Appl. Environ. Microbiol..

[14] Gravesen A (2002). High-level resistance to class IIa bacteriocins is associated with one general mechanism in *Listeria monocytogenes*. Microbiol. Read. Engl..

[15] Dalet K, Cenatiempo Y, Cossart P, Héchard Y (2001). & European Listeria Genome Consortium. A sigma(54)-dependent PTS permease of the mannose family is responsible for sensitivity of *Listeria monocytogenes* to mesentericin Y105. Microbiol. Read. Engl..

[16] Héchard Y, Pelletier C, Cenatiempo Y, Frère J (2001). Analysis of sigma(54)-dependent genes in *Enterococcus faecalis*: a mannose PTS permease (EII(Man)) is involved in sensitivity to a bacteriocin, mesentericin Y105. Microbiol. Read. Engl..

[17] Ramnath M, Arous S, Gravesen A, Hastings JW, Héchard Y (2004). Expression of *mptC* of *Listeria monocytogenes* induces sensitivity to class IIa bacteriocins in *Lactococcus lactis*. Microbiol. Read. Engl..

[18] Kjos M, Salehian Z, Nes IF, Diep DB (2010). An extracellular loop of the mannose phosphotransferase system component IIC is responsible for specific targeting by class IIa bacteriocins. J. Bacteriol..

[19] Tosukhowong A (2012). Garvieacin Q, a novel class II bacteriocin from *Lactococcus garvieae* BCC 43578. Appl. Environ. Microbiol..

[20] Raya R, Bardowski J, Andersen PS, Ehrlich SD, Chopin A (1998). Multiple transcriptional control of the *Lactococcus lactis trp* operon. J. Bacteriol..

[21] Gasteiger E (2003). ExPASy: the proteomics server for in-depth protein knowledge and analysis. Nucleic Acids Res..

[22] McWilliam H (2013). Analysis Tool Web Services from the EMBL-EBI. Nucleic Acids Res..

[23] Corpet F (1988). Multiple sequence alignment with hierarchical clustering. Nucleic Acids Res..

[24] Tusnády GE, Simon I (2001). The HMMTOP transmembrane topology prediction server. Bioinforma. Oxf. Engl..

[25] Omasits U, Ahrens CH, Müller S, Wollscheid B (2014). Protter: interactive protein feature visualization and integration with experimental proteomic data. Bioinforma. Oxf. Engl..

[26] Biasini M (2014). SWISS-MODEL: modelling protein tertiary and quaternary structure using evolutionary information. Nucleic Acids Res..

[27] Neidhardt, F. C., Ingraham, J. L. & Schaechter, M. *Physiology of the bacterial cell: a molecular approach* (Sinauer Associates Inc, 1990).

[28] Sedlacek I, Benda P (1998). Isolation of *Lactococcus garvieae* species from bovine mastitis. Veterinární Medicína.

[29] Haghighi Karsidani S, Soltani M, Nikbakhat-Brojeni G, Ghasemi M, Skall H (2010). Molecular epidemiology of zoonotic streptococcosis/lactococcosis in rainbow trout (*Oncorhynchus mykiss*) aquaculture in Iran. Iran. J. Microbiol..

[30] Villani F (2001). Detection and characterization of a bacteriocin, garviecin L1-5, produced by *Lactococcus garvieae* isolated from raw cow’s milk. J. Appl. Microbiol..

[31] Borrero J (2011). Characterization of garvicin ML, a novel circular bacteriocin produced by *Lactococcus garvieae* DCC43, isolated from mallard ducks (*Anas platyrhynchos*). Appl. Environ. Microbiol..

[32] Maldonado-Barragán A (2013). Garvicin A, a novel class IId bacteriocin from *Lactococcus garvieae* that inhibits septum formation in *L*. *garvieae* strains. Appl. Environ. Microbiol..

[33] Gao Y, Li D, Liu S, Zhang L (2015). Garviecin LG34, a novel bacteriocin produced by *Lactococcus garvieae* isolated from traditional Chinese fermented cucumber. Food Control.

[34] Ovchinnikov, K. V. *et al*. A novel group of leaderless and multi-peptide bacteriocins from gram-positive bacteria. *Appl. Environ. Microbiol*. AEM.01094-16 doi:10.1128/AEM.01094-16 (2016). 10.1128/AEM.01094-16 PMC4988205 27316965

[35] Wyszyńska A, Raczko A, Lis M, Jagusztyn-Krynicka EK (2004). Oral immunization of chickens with avirulent *Salmonella* vaccine strain carrying *C*. *jejuni* 72Dz/92 *cjaA* gene elicits specific humoral immune response associated with protection against challenge with wild-type *Campylobacter*. Vaccine.

[36] Korlath JA, Osterholm MT, Judy LA, Forfang JC, Robinson RA (1985). A point-source outbreak of campylobacteriosis associated with consumption of raw milk. J. Infect. Dis..

[37] Fonzi WA, Irwin MY (1993). Isogenic strain construction and gene mapping in *Candida albicans*. Genetics.

[38] Koryszewska-Bagińska, A., Aleksandrzak-Piekarczyk, T. & Bardowski, J. Complete genome sequence of the probiotic strain *Lactobacillus casei* (formerly *Lactobacillus paracasei*) LOCK919. *Genome Announc*. **1** (2013). 10.1128/genomeA.00758-13 PMC3784782 24072862

[39] Axelsson L (2012). Genome sequence of the naturally plasmid-free *Lactobacillus plantarum* strain NC8 (CCUG 61730). J. Bacteriol..

[40] Kleerebezem M (2003). Complete genome sequence of *Lactobacillus plantarum* WCFS1. Proc. Natl. Acad. Sci. USA.

[41] Aleksandrzak-Piekarczyk, T., Koryszewska-Baginska, A. & Bardowski, J. Genome sequence of the probiotic strain *Lactobacillus rhamnosus* (formerly *Lactobacillus casei*) LOCK900. *Genome Announc*. **1** (2013). 10.1128/genomeA.00640-13 PMC3744693 23950137

[42] Koryszewska-Baginska, A., Bardowski, J. & Aleksandrzak-Piekarczyk, T. Genome sequence of the probiotic strain *Lactobacillus rhamnosus* (formerly *Lactobacillus casei*) LOCK908. *Genome Announc*. **2** (2014). 10.1128/genomeA.00120-14 PMC3931371 24558250

[43] Morita H (2009). Complete genome sequence of the probiotic *Lactobacillus rhamnosus* ATCC 53103. J. Bacteriol..

[44] Holck A, Axelsson L, Birkeland SE, Aukrust T, Blom H (1992). Purification and amino acid sequence of sakacin A, a bacteriocin from *Lactobacillus sake* Lb706. J. Gen. Microbiol..

[45] Kobierecka P (2015). Lactic acid bacteria as a surface display platform for *Campylobacter jejuni* antigens. J. Mol. Microbiol. Biotechnol..

[46] Bolotin A (2001). The complete genome sequence of the lactic acid bacterium *Lactococcus lactis* ssp. *lactis* IL1403. Genome Res..

[47] Glaser P (2001). Comparative genomics of *Listeria* species. Science.

[48] Nieto Lozano JC, Meyer JN, Sletten K, Peláz C, Nes IF (1992). Purification and amino acid sequence of a bacteriocin produced by *Pediococcus acidilactici*. J. Gen. Microbiol..

[49] Chumley FG, Menzel R, Roth JR (1979). Hfr formation directed by Tn10. Genetics.

[50] Kleerebezem M, Beerthuyzen MM, Vaughan EE, Vos WMde, Kuipers OP (1997). Controlled gene expression systems for lactic acid bacteria: transferable nisin-inducible expression cassettes for *Lactococcus*, *Leuconostoc*, and *Lactobacillus* spp. Appl. Environ. Microbiol..

[51] de Ruyter PG, Kuipers OP, de Vos WM (1996). Controlled gene expression systems for *Lactococcus lactis* with the food-grade inducer nisin. Appl. Environ. Microbiol..

