# Supplementary material for: The Non-Lantibiotic Bacteriocin Garvicin Q Targets Man-PTS in a Broad Spectrum of Sensitive Bacterial Genera
Source: Sci Rep. 2017 Aug 21;7:8359. doi: 10.1038/s41598-017-09102-7 (PMC5566476; doi:10.1038/s41598-017-09102-7)
Supplement: Supplementary file 1 — Supplementary Table S1 & Figure S1 [file 41598_2017_9102_MOESM1_ESM.pdf]

The Non-Lantibiotic Bacteriocin Garvicin Q Targets Man-PTS in a Broad Spectrum of Sensitive Bacterial Genera

ALEKSANDRA TYMOSZEWSKA[1], DZUNG B. DIEP[2], PAULINA WIRTEK[1] AND TAMARA ALEKSANDRZAK-PIEKARCZYK[1*]

[1]Institute of Biochemistry and Biophysics, Polish Academy of Sciences (IBB PAS), Pawińskiego 5a, 02-106 Warsaw, Poland

[2]Faculty of Chemistry, Biotechnology and Food Science, Norwegian University of Life Sciences, Ås, Norway

*Corresponding author. Mailing address: IBB PAS, Pawińskiego 5a, 02-106 Warsaw, Poland, Phone: (4822) 592 1213, Fax: (4822) 658 4636, E-mail: tamara@ibb.waw.pl

Table S1. Bacterial strains, plasmids and primers used in this study

Strain[a], primer	Description, NCBI accession number, primer sequence[b]	Source and/or reference
Strains		
Bacillus cereus IBB3390	indicator strain	IBB PAS
Bacillus subtilis 168	indicator strain	IBB PAS
Campylobacter jejuni 12	indicator strain	BUW[35]
Campylobacter jejuni 480	indicator strain	BUW
Campylobacter jejuni 81176	indicator strain	BUW[36]
Campylobacter coli 23	indicator strain	BUW
Candida albicans CAI-4	indicator strain	IBB PAS[37]
Carnobacterium maltaromaticum IBB3447	indicator strain	IBB PAS
Enterococcus durans IBB3441	indicator strain	IBB PAS
Enterococcus faecalis IBB3439	indicator strain	IBB PAS
Enterococcus faecalis IBB3444	indicator strain	IBB PAS
Enterococcus faecalis LMGT 2003	indicator strain	LMGT NMBU
Enterococcus faecium LMGT 2783	indicator strain	LMGT NMBU
Enterococcus faecium LMGT 2787	indicator strain	LMGT NMBU
Lactobacillus casei IBB3418	indicator strain	IBB PAS
Lactobacillus casei IBB3427	indicator strain	IBB PAS
Lactobacillus casei LOCK 0919	indicator strain, CP005486.1	LOCK[38]
Lactobacillus casei IBB3423	indicator strain	IBB PAS
Lactobacillus casei/paracasei IBB3425	indicator strain	IBB PAS
Lactobacillus casei/paracasei IBB3426	indicator strain	IBB PAS
Lactobacillus casei/paracasei IBB3428	indicator strain	IBB PAS
Lactobacillus paracasei IBB3424	indicator strain	IBB PAS
Lactobacillus johnsonii IBB3155	indicator strain	IBB PAS
Lactobacillus kunkeei AH1	indicator strain	LMGT NMBU
Lactobacillus kunkeei AH38	indicator strain	LMGT NMBU
Lactobacillus kunkeei AH119	indicator strain	LMGT NMBU
Lactobacillus paraplantarum IBB3438	indicator strain	IBB PAS
Lactobacillus plantarum NC8	indicator strain, AGRI00000000.1	LMGT NMBU[39]
Lactobacillus plantarum WCSF1	indicator strain, AL935263.2	LMGT NMBU[40]
Lactobacillus plantarum IBB3036	indicator strain	IBB PAS
Lactobacillus plantarum IBB3433	indicator strain	IBB PAS
Lactobacillus plantarum IBB3436	indicator strain	IBB PAS
Lactobacillus plantarum IBB3434	indicator strain	IBB PAS
Lactobacillus rhamnosus IBB3429	indicator strain	IBB PAS
Lactobacillus rhamnosus LOCK 0900	indicator strain, CP005484.1	LOCK[41]
Lactobacillus rhamnosus LOCK 0908	indicator strain, CP005485.1	LOCK[42]
Lactobacillus rhamnosus GG	indicator strain, AP011548	Dicoflor (Vitis Pharma, Poland)[43]
Lactobacillus sake Lb706	sakacin A producer	LMGT NMBU[44]
Lactobacillus salivarius IBB3154	indicator strain	IBB PAS[45]
Lactococcus garvieae IBB3403	indicator strain	IBB PAS
Lactococcus garvieae IBB66	indicator strain	IBB PAS
Lactococcus lactis IBB3404	indicator strain	IBB PAS
Lactococcus lactis IBB3411	indicator strain	IBB PAS
Lactococcus lactis B190 LMGT 2130	LcnA producer	LMBT NMBU[7]
Lactococcus lactis QU5 LMGT 3419	indicator strain	LMBT NMBU
Lactococcus lactis subsp. *cremoris* IBB3409	indicator strain	IBB PAS
Lactococcus lactis subsp. *lactis* IL1403	indicator strain, AE005176	IBB PAS[46]
Lactococcus lactis subsp. *lactis* IBB2955	indicator strain	IBB PAS
Lactococcus lactis subsp. *lactis* IBB3407	indicator strain	IBB PAS
Lactococcus raffinolactis IBB91	indicator strain	IBB PAS
Leuconostoc lactis IBB3446	indicator strain	IBB PAS
Leuconostoc mesenteroides IBB3442	indicator strain	IBB PAS
Leuconostoc mesenteroides IBB3443	indicator strain	IBB PAS
Listeria monocytogenes EGD-e LMGT 2604	indicator strain, AL591824.1	LMGT NMBU[47]

Pediococcus acidilactici LMGT 2002	indicator strain	LMGT NMBU
Pediococcus acidilactici LMGT 2351	pediocin PA-1 producer	LMGT NMBU[48]
Pediococcus parvulus IBB3448	indicator strain	IBB PAS
Pseudomonas aeruginosa ATCC 9027	indicator strain	ATCC
Salmonella typhimurium TT622	indicator strain	AMUW[49]
Staphylococcus aureus ATCC 6538	indicator strain	ATCC
Staphylococcus caprae DSM-20608	indicator strain	DSMZ
Staphylococcus delphini DSM-20771	indicator strain	DSMZ
Staphylococcus epidermidis DSM-20044	indicator strain	DSMZ
Staphylococcus hyicus DSM-20459	indicator strain	DSMZ
Staphylococcus intermedius DSM-20373	indicator strain	DSMZ
Staphylococcus lugdunesis DSM-4804	indicator strain	DSMZ
Staphylococcus pseudintermedius DSM-21284	indicator strain	DSMZ
Staphylococcus saprophyticus DSM-18669	indicator strain	DSMZ
Staphylococcus schleiferi DSM-6628	indicator strain	DSMZ
Streptococcus agalactiae IBB123	indicator strain	IBB PAS
Streptococcus agalactiae IBB130	indicator strain	IBB PAS
Streptococcus mitis IBB3449	indicator strain	IBB PAS
Streptococcus sobrinus IBB3450	indicator strain	IBB PAS
Streptococcus parauberis IBB272	indicator strain	IBB PAS
Lactococcus lactis IL1403 - spontaneous mutants		
LMGT3726	LcnA-resistant	LMBT NMBU[10]
LMGT3727	LcnA-resistant	LMBT NMBU
Lactococcus lactis IL1403 - other mutants		
B464	*ptnABCD* operon deletion IL1403 mutant	LMBT NMBU[7]
B488	B464 with pNZ9530	LMBT NMBU[7]
B520	B464 with pNZ9530 and pNZ8037	LMBT NMBU[7]
B515	B488 with pNZ8037 with *ptnABCD*	LMBT NMBU[7]
B529	B488 with pNZ8037 with *ptnCD*	LMBT NMBU[7]
B538	B488 with pNZ8037 with *ptnC*	LMBT NMBU[7]
B541	B488 with pNZ8037 with *ptnD*	LMBT NMBU[7]
B542a	B529 with Pro102His substitution in *ptnC*	This study
B543a	B529 with Ala104Val substitution in *ptnC*	This study
B544a	B529 with Pro108Ser substitution in *ptnD*	This study
B545a	B529 with Thr120Ile substitution in *ptnD*	This study
B546a	B529 with Gly244Val substitution in *ptnD*	This study
B530a	B488 with pNZ8037 with *manCD*	This study
B547a	*manCD* strain with Pro126His substitution in *manD*	This study
Escherichia coli EC1000		
B529a	strain with pNZ8037 harbouring *ptnCD*	This study
B542a	*ptnCD* strain with Pro102His substitution in *ptnC*	This study
B543a	*ptnCD* strain with Ala104Val substitution in *ptnC*	This study
B544a	*ptnCD* strain with Pro108Ser substitution in *ptnD*	This study
B545a	*ptnCD* strain with Thr120Ile substitution in *ptnD*	This study
B546a	*ptnCD* strain with Gly244Val substitution in *ptnD*	This study
B530a	strain with pNZ8037 harbouring *manCD*	This study
B547a	*manCD* strain with Pro126His substitution in *manD*	This study
Plasmids		
pNZ9530	Cam^r, containing nisin-regulatory genes *nisRK*	[50]
pNZ8037	Em^r, containing nisin-responsive promoter	[51]
Primers	DNA sequence (5'→3')	
*manC*for/rev	CGTGATCTCGGCGTTA/TAACGCTCAAGCGTGTG	
*manD*for/rev	CGCTCTTATCTACCTC/GCCAATTTAGTGCTCCTAAC	
*ptnC*for/rev	TCTGACCTCTTTGGTTTG/GCATACGTTCGTAGT	
*ptnD*for/rev	AACCTCCAAGCTTCTG/AGCCACAGATTCTCTCC	
27F/1492R	AGAGTTTGATCMTGGCTCAG/GGTTACCTTGTTACGACTT	
*ptnC*Pro102Hisfor/rev	CATGGGTACTATCGTTCATGCTGCTATCTTGCTTG/CAAGCAAGATAGCAGCATG AACGATAGTACCCATG	
*ptnC*Ala104Valfor/rev	GTACTATCGTTCCTGCTGTTATCTTGCTTGCAACTGC/GCAGTTGCAAGCAAGAT AACAGCAGGAACGATAGTA	

*ptnD*Pro108Serfor/rev	AGTTGGTATGATGGGTTCTCTTGCCGGTATCGG/CCGATACCGGCAAGAGAACCCATCATACCAACT
*ptnD*Thr120Ilefor/rev	GGTGACCCTGTCTTCTGGTTTATAGTACGTCCTATC/GATAGGACGTACTATAAACCAGAAGACAGGGTCACC
*ptnD*Gly244Valfor/rev	TCATGATATTCTTGGTCAAGTTGTTAACAAACTTTCTCTTGATCCTA/TAGGATCAAGAGAAAGTTTGTTAACAACTTGACCAAGAATATCATGA
compl*manCD*for/rev	TAGCCCATGGATGAGTATTATTTCAATCATTTTAG/ATGCCTCGAGTTATTAAGCAAGACCTGCAACGT
*manD*Pro126Hisfor/rev	CTGGTTCACAGTACGTCATATCGTTGGTGCCATCG/CGATGGCACCAACGATATGACGTACTGTGAACCAG

[a] Bacterial strains are derived from the Regional Strains and Plasmids Collection of the Institute of Biochemistry and Biophysics, Warsaw, Poland (IBB PAS); from the collection of the Faculty of Biology, University of Warsaw, Warsaw, Poland (BUW); from the collection of the Department of Applied Microbiology, Faculty of Biology, University of Warsaw (AMUW); from the Pure Cultures Collection of the Institute of Fermentation Technology and Microbiology, Technical University of Lodz, Poland (LOCK); from the collection of the Laboratory of Microbial Gene Technology, Department of Chemistry, Biotechnology and Food Science, Norwegian University of Life Sciences, Ås, Norway (LMGT NMBU); from the Collection of Microorganisms and Cell Cultures, Germany (DSMZ); from the American Type Culture Collection (ATCC) or were obtained in this study.
[b] Cam^r indicates chloramphenicol resistance, Em^r indicates erythromycin resistance. Restriction sites added to primers are underlined (NcoI; XhoI).

Figure S1. Alignment of Man-PTS IIC (a) and IID (b) transmembrane subunits. Outside, transmembrane and inside regions are indicated with red, black and green letters, respectively. Region α in IIC and region γ in IID are underlined. Amino acid substitutions (missense mutations) introduced by spontaneous mutation or by site-directed mutagenesis are highlighted grey or green, respectively , and their counterparts in other species are boxed. Asterisks indicate fully conserved residues, double dots - strongly conserved residues, single dots - weakly conserved residues.

(a)

```
L. garvieae IBB3403         60 WANVGAAVAPDAALASVASAILMVQGGNFDLTHITGVIVPAAILLATAGLVLTTLV 117
L. garvieae IBB66           60 WANVGAAVAPDAALASVASAILMVQGGNFDLTHITGVIVPAAILLATAGLVLTTLV 117
L. lactis IBB3409           62 WANVGAAVAPDAALASIASSILMVQSNNFDLTHIMGTIVPAAILLATAGLVLTTLV 119
L. lactis IBB3407           62 WANVGAAVAPDAALASVASSILMVQSNNFDLTHIMGTIVPAAILLATAGLVLTTLV 119
L. lactis IL1403            62 WANVGAAVAPDAALASIASSILMVQSNNFDLTHIMGTIVPAAILLATAGLVLTTLV 119
L. lactis IBB2955           62 WANVGAAVAPDAALASVASSILMVQSNNFDLTHIMGTIVPAAILLATAGLVLTTLV 119
L. lactis LMGT3419          62 WANVGAAVAPDAALASVASSILMVQSNNFDLTHIMGTIVPAAILLATAGLVLTTLV 119
L. rhamnosus GG             62 WANIGAAVAPDAALASVASAIILVLGGQGTKG--VSSAIAIAVPLAVAGLLLTTLV 117
L. rhamnosus IBB3429        62 WANIGAAVAPDAALASVASAIILVLGGQGTKG--VSSAIAIAVPLAVAGLLLTTLV 117
L. rhamnosus LOCK 0900      62 WANIGAAVAPDAALASVASAIILVLGGQGTKG--VSSAIAIAVPLAVAGLLLTTLV 117
L. rhamnosus LOCK 0908      62 WANIGAAVAPDAALASVASAIILVLGGQGTKG--VSSAIAIAVPLAVAGLLLTTLV 117
L. casei IBB3418            62 WANIGAAVAPDAALASVASAIILVLGGQGTKG--ISSAIAIAVPLAVAGLLLTTLV 117
L. casei LOCK 0919          62 WANIGAAVAPDAALASVASAIILVLGGQGTKG--ISSAIAIAVPLAVAGLLLTTLV 117
L. paracasei IBB3424        62 WANIGAAVAPDAALASVASAIILVLGGQGTKG--ISSAIAIAVPLAVAGLLLTTLV 117
L. casei IBB3427            62 WANIGAAVAPDAALASVASAIILVLGGQGTKG--ISSAIAIAVPLAVAGLLLTTLV 117
L. plantarum IBB3036        62 WANIGAAVAPDAALASIASAIILVLGGQGRAG--VTSAIAIAVPLAVAGLLLTIIA 117
L. plantarum IBB3434        62 WANIGAAVAPDAALASIASAIILVLGGQGRAG--VTSAIAIAVPLAVAGLLLTIIA 117
L. plantarum NC8            62 WANIGAAVAPDAALASIASAIILVLGGQGRAG--VTSAIAIAVPLAVAGLLLTIIA 117
L. curvatus IBB3438         62 WSNVGAAVAPDAALAAIASAIILVLGGQGRAG--VSSAIAIAVPLAVAGLLLTILA 117
L. plantarum IBB3436        62 WSNVGAAVAPDAALAAIASAIILVLGGQGRAG--VSSAIAIAVPLAVAGLLLTILA 117
L. plantarum WCFS1          62 WSNVGAAVAPDAALAAIASAIILVLGGQGRAG--VSSAIAIAVPLAVAGLLLTILA 117
L. salivarius IBB3154       60 WANIGAAVAPDAALAAVASAIILVKGGQGTAG--ISTAIAVAIPLAVAGLFLTMIV 115
E. faecium LMGT2783         60 WANIGAAVAPDAALASVASAIILVLGGQGVKG--VPSAIAIAVPLAVAGLFLTMIV 115
L. monocytogenes EGD-e      60 WANIGAAVAPDAALASVASAIILVLGGQGVAG--IPSAIAIAIPLAVAGLFLTMIV 115
                               *:*:***********::**:*::* . :          :  *: **.***.** :.
```

(b)

```
L. garvieae IBB3403         77 PIVGVTLALEEEIANGVEIDEAAIQGVKVGMMGPLAGIGDPVFWFTVRPIVGAIAA 134
L. garvieae IBB66           77 PIVGVTLALEEEIANGVEIDEAAIQGVKVGMMGPLAGIGDPVFWFTVRPIVGAIAA 134
L. lactis IBB3409           74 PIIGVTLALEEERANGADIDDAAIQGVKVGMMGPLAGIGDPVFWFTVRPIVGAIAA 131
L. lactis IL1403            74 PIIGVTLALEEERANGADIDDAAIQGVKVGMMGPLAGIGDPVFWFTVRPIVGAIAA 131
L. lactis IBB2955           74 PIIGVTLALEEERANGADIDDAAIQGVKVGMMGPLAGIGDPVFWFTVRPIVGAIAA 131
L. lactis IBB3407           74 PIIGVTLALEEERANGADIDDAAIQGVKVGMMGPLAGIGDPVFWFTVRPIVGAIAA 131
L. lactis LMGT3419          74 PIIGVTLALEEERANGADIDDAAIQGVKVGMMGPLAGIGDPVFWFTVRPIVGAIAA 131
L. curvatus IBB3438         74 PIIGVTMALEEERANGAPIDDVTIQGVKVGMMGPLAGVGDPVFWFTLKPIIGALAA 131
L. plantarum IBB3436        74 PIIGVTMALEEERANGAPIDDVTIQGVKVGMMGPLAGVGDPVFWFTLKPIIGALAA 131
L. plantarum WCFS1          74 PIIGVTMALEEERANGAPIDDVTIQGVKVGMMGPLAGVGDPVFWFTLKPIIGALAA 131
L. plantarum IBB3036        79 PVIGVTLALEEERANGAPIDDVTIQGVKVGMMGPLAGVGDPVFWYTVKPIVGALAA 136
L. plantarum NC8            79 PVIGVTLALEEERANGAPIDDVTIQGVKVGMMGPLAGVGDPVFWYTVKPIVGALAA 136
L. plantarum IBB3434        79 PVIGVTLALEEERANGAPIDDVTIQGVKVGMMGPLAGVGDPVFWYTVKPIVGALAA 136
L. rhamnosus IBB3429        73 PILGVTMALEEERANGAPIDDKAIQGVKVGMMGPLAGVGDPVFWFTVKPIIGALAA 130
L. rhamnosus LOCK0900       73 PILGVTMALEEERANGAPIDDKAIQGVKVGMMGPLAGVGDPVFWFTVKPIIGALAA 130
L. rhamnosus LOCK0908       73 PILGVTMALEEERANGAPIDDKAIQGVKVGMMGPLAGVGDPVFWFTVKPIIGALAA 130
L. rhamnosus GG             73 PILGVTMALEEERANGAPIDDKAIQGVKVGMMGPLAGVGDPVFWFTVKPIIGALAA 130
L. casei IBB3427            73 PIIGVTMALEEERANGAPIDDKAIQGVKVGMMGPLAGVGDPVFWFTVKPIIGALAA 130
L. casei IBB3418            73 PIIGVTMALEEERANGAPIDDKAIQGVKVGMMGPLAGVGDPVFWFTVKPIIGALAA 130
L. casei LOCK0919           73 PIIGVTMALEEERANGAPIDDKAIQGVKVGMMGPLAGVGDPVFWFTVKPIIGALAA 130
L. paracasei IBB3424        73 PIIGVTMALEEERANGAPIDDKAIQGVKVGMMGPLAGVGDPVFWFTVKPIIGALAA 130
L. salivarius IBB3154       74 PILGVTLALEEDRANGAPVDDVAINGVKVGMMGPLAGVGDPVFWFTARPIIGALGA 131
E. faecium LMGT2783         74 PILGVTLALEEERANGAPVDDVAIQGVKVGMMGPLAGVGDPVFWFTVRPMLGALGA 131
L. monocytogenes EGD-e      73 PILGVTLALEEERANGAEVDDVAIQGVKVGMMGPLAGVGDPVFWFTIRPMLGALGA 130
                               *::***:****: ***. :*: :*:************:******:* :*::**:.*
```

```
IBB3403    223 GAYVGEWIDKAGKVVVQGAQTGTTGDGVAKFDWLDQAGNVVGNGVAGQGGFAHYVTVDQLNTVDGSTLHNILGQVSSG--LGLSPEQTQSLQDVFNS 318
IBB66      223 GAYVGEWIDKAGKVVVQGAQTGTTGDGVAKFDWLDQAGNVVGNGVAGQDGFAHYVTVDQLNTVDGSTLHNILGQVSSG--LGLSPEQTQSLQDVFNS 318
IBB3409    220 GAYVE-FPKG---------------------------------------------------SVSGTQLHEILGQV--GNKLSLDPTKVTYLQDNLNQ 263
IL1403     220 GAYLE-FPKG---------------------------------------------------SVSGTQLHDILGQV--GNKLSLDPTKVTYLQDNLNQ 263
IBB2955    220 GAYVE-FPKG---------------------------------------------------SVSGTQLHDILGQV--GNKLSLDPTKVTYLQDNLNQ 263
IBB3407    220 GAYVE-FPKG---------------------------------------------------SVSGTQLHDILGQV--GNKLSLDPTKVTYLQDNLNQ 263
LMGT3419   220 GAYVE-FPKG---------------------------------------------------SVSGTQLHDILGQV--GNKLSLDPTKVTYLQDNLNQ 263
IBB3438    218 GAYID-WDKL---------------------------------------------------PKGAAGIKEALTQQAAG--RSLDKYKVTTLQDNLNQ 261
IBB3436    218 GAYID-WDKL---------------------------------------------------PKGAAGIKEALTQQAAG--RSLDKYKVTTLQDNLNQ 261
WCFS1      218 GAYID-WDKL---------------------------------------------------PKGAAGIKEALTQQAAG--RSLDKYKVTTLQDNLNQ 261
IBB3036    223 GSYID-WDKL---------------------------------------------------PKGASGVKEALTQQAAG--RSLDKYKVTTLQDNLNQ 266
NC8        223 GSYID-WDKL---------------------------------------------------PKGASGVKEALTQQAAG--RSLDKYKVTTLQDNLNQ 266
IBB3434    223 GSYID-WDKL---------------------------------------------------PKGASGVKEALTQQAAG--RSLDKYKVTTLQDNLDQ 266
IBB3429    216 GAYID-WSSL---------------------------------------------------PSGKAGVQKALELQSQG--LSLTKNKVTTLQDNLDS 259
LOCK0900   216 GAYID-WSSL---------------------------------------------------PSGKAGVQKALELQSQG--LSLTKNKVTTLQDNLDS 259
LOCK0908   216 GAYID-WSSL---------------------------------------------------PSGKAGVQKALELQSQG--LSLTKNKVTTLQDNLDS 259
GG         216 GAYID-WSSL---------------------------------------------------PSGKAGVQKALELQSQG--LSLTKNKVTTLQDNLDS 259
IBB3427    216 GAYID-WSSI---------------------------------------------------PNGTKGIQKALELQAQG--LSLTKNKITTLQDNLDS 259
IBB3418    216 GAYID-WSSL---------------------------------------------------PSGSKGIQKALELQSQG--LSLTKNKVLTLQDNLDS 259
LOCK0919   216 GAYID-WSSL---------------------------------------------------PSGSKGIQKALELQSQG--LSLTKNKVLTLQDNLDS 259
IBB3424    216 GAYID-WSSL---------------------------------------------------PSGSKGIQKALELQSQG--LSLTKNKVLTLQDNLDS 259
IBB3154    217 GGYID-WDHI---------------------------------------------------PSGAKGIKEVLTQWNMGKGMSLDKIKVTTLQDNLDQ 262
LMGT2783   217 GAYIE-WDKL---------------------------------------------------PSGGEGIKSAFEQVNNG--LALSPEKVTTLQDNLDQ 260
EGD-e      216 GAYID-WSHL---------------------------------------------------PQGAQGIKTALEQQQAG--LALSEIKVTTLQNNLDN 259
               *.*:  :                                                            ::  :     *   .*   :   **: ::.
```
